# Polymer extrusion filter remaining useful life dataset from contamination experiments

**DOI:** 10.1016/j.dib.2026.113254

**Published:** 2026-09-08

**Authors:** Corentin Ferreira, Ben Ko, Wen Li, Kevin Otto

**Affiliations:** Department of Mechanical Engineering, The University of Melbourne, 3010 Parkville, Victoria, Australia

**Keywords:** Time-series, Remaining useful life, Classification, Prognostics, Predictive maintenance, Manufacturing

## Abstract

Predictive Maintenance has become an essential objective in industry as it has the potential to decrease unplanned downtime, reduce inventories of replacement parts and increase operational safety, by identifying and predicting the time of failures. To develop accurate predictive maintenance systems, sufficient historical data to failure must be acquired. While such datasets are publicly available for components (i.e. bearing and motors), their public availability for industrial manufacturing systems is limited. This article presents a time-series dataset for the run-to-failure of single-screw polymer extruder filters. The dataset includes the temperatures along the barrel of the extruder, screw motor voltage and current at 1 Hz. The current intake of the extruder is also provided and acquired at 1 kHz, to support feature engineering works. Data is collected for 60 run-to-failures during the extrusion of low-density polyethylene. Two failure modes were recorded: full filter clogging, preventing the extrusion of the material, and filter failure, where the material accumulates until the filter bursts. The established dataset can be used to develop and verify state-of-the-art predictive maintenance models under industrial conditions, physics-based models, feature engineering and multi-rate data fusion methodologies.

Specifications Table

**Table:** 

Subject	Engineering & Materials science
Specific subject area	Single-screw polymer extruder filter prognosis
Type of data	Table (.csv format) Raw
Data collection	The dataset was acquired on a single-screw polymer extruder, which compounds polymer granules into a melt which is pushed through a die to shape the final product. During granule production, handling and storage, contaminants enter the feedstock material. These contaminants are filtered in the die by a stainless-steel mesh filter requiring periodic replacement to ensure material flow and purity. During the extrusion process: - Extruder feed, back, middle and front temperatures are recorded off thermocouples type-K, - Screw motor voltage and current were collected using a current shunt, - Extruder current draw is collected using a current clamp. Filter material: 304 Stainless-steel mesh, 80 openings per inch Contaminant: Calcite (CaCO3) Extruded Polymer: ExxonMobil LLDPE 6101
Data source location	Institution: The University of Melbourne City, State, Country: Parkville, Victoria, Australia
Data accessibility	Repository name: Zenodo Data identification number: 10.5281/zenodo.21,987,832 Direct URL to data: https://zenodo.org/records/21987832
Related research article	None

## 1 Value of the Data

• This dataset provides complete run-to-failure records of polymer extrusion filters during operation. The extrusion operation is performed in an industrial environment, enabling the development and evaluation of Remaining Useful Life (RUL) and prognostic methods, for example through the construction of degradation sensitive health indicators. Process temperatures and screw motor variables were acquired at 1 Hz, while extruder current draw was acquired at 1 kHz, making the dataset suitable to investigate multi-rate data fusion, signal processing and feature engineering for industrial health monitoring, as public datasets for the degradation of industrial assets are scarce [1].
• The availability of 60 degradation runs across two failure modes supports research into failure mode classification (isolating the failure mode), RUL regression and prognostics (where both failure mode and associated remaining life are estimated). The data was acquired under industrial operating conditions and retains process and measurement variability present during the experiments. Failure modes are imbalanced, with notably less filter bursts than filter clogs, reflecting real industrial challenges. To achieve such research objectives, the data is fit for use with machine learning methods.
• The high-frequency extruder current signal enables researchers to extract time-domain (windowed-means, -standard deviations and other statistics), frequency-domain (Fourier Transform) and time-frequency (wavelet decompositions) features that may not be observable from low-frequency process measurements alone. This supports comparative studies on the diagnostic and prognostic value of conventional process variables versus high-frequency electrical signatures.
• Researchers and practitioners in predictive maintenance or prognostics, smart manufacturing and polymer processing can reuse this dataset to study sensor and feature selection, model performance and generalization, without the need to acquire costly and time-consuming RUL data.

## 2 Background

Polymer extrusion processes often use filtration systems to remove contaminants, gels, degraded material or other unwanted particles from the polymer melt before downstream processing [2]. During operations, the filters progressively degrade or become obstructed, which eventually requires filter replacement or cleaning [3]. As production is impacted by filter changes, it is desirable to predict when a filter will require intervention, so as to minimally interrupt production.

This dataset was compiled to record the operational behaviour of polymer extrusion filters from normal, healthy operations through to end-of-life under industrial conditions. The dataset was generated from 60 run-to-failure experiments, during which two failure modes were observed: full filter clogging or filter burst. To accelerate the testing, the experimental procedure provided by Schöppner & Meilwes [4] was adopted. Measurements include process temperatures, screw motor voltage and screw motor current nominally sampled at 1 Hz, together with extruder total current draw nominally sampled at 1 kHz.

The dataset was prepared as a standalone data article to provide detailed access to the raw run-to-failure records, sensor channels, sampling rates and failure labels. Related research in extrusion [3,5,6] has not publicly disclosed their RUL data, and on a broader scope, industrial systems RUL data is rarely available [7]. Literature on how to conduct RUL modelling for industrial cases and its challenges is available in Benhanifia et al. [8] and Huang et al. [9]. As an extruder possesses a central rotating element, methods provided in Zhou et al. [10,11] can also apply here and could inform users of this dataset on how to build informative health indicators.

## 3 Data Description

**Fig. 1:**
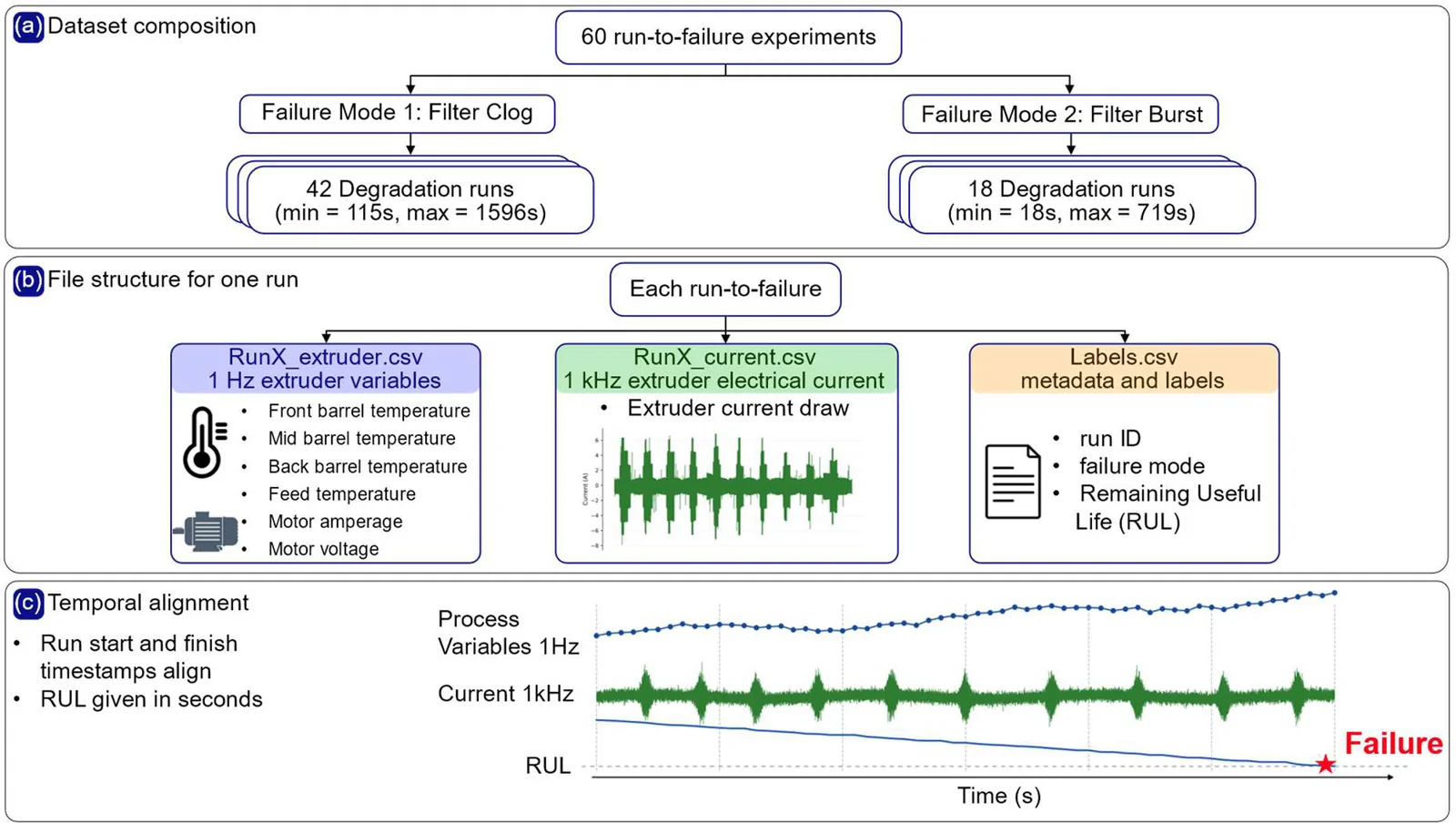
Structure of the polymer extrusion filter run-to-failure dataset. (a) Distribution of the 60 degradation runs across the two observed failure modes. (b) Repository structure, showing the low-frequency process variable files, high-frequency current file.

The dataset consists of 60 runs-to-failure records collected from polymer extrusion filters during operation in an industrial environment, where other plant items operate at the same time (CNCs, welding stations, peripherals, heating system). Each record corresponds to one degradation run of a filter and contains process measurements (temperatures, screw motor voltage and amperage), high-frequency extruder electrical current measurements and run-level labels (failure mode and total filter lifetime). The dataset includes two imbalanced failure modes: 42 runs correspond to a full filter clogging (no more material throughput) and 18 runs correspond to filter bursts (the mesh cracks under the mechanical load from the contaminants). For both failure modes, the contaminant accumulation and progressive filter loading constitute a common degradation process, clogging and filter burst representing two competing terminal outcomes of the degradation process. The released labels retain the terminal outcome for each trajectory so that users may formulate the data problem as a competing outcome or as an outcome-specific classification problem. Recorded lifetimes for the filter bursts range from 18 s to 719 s. The clogging lifetimes range from 115 s to 1596 s. In total, 27 801 samples at 1 Hz and 27 801 000 samples at 1 kHz are available across all runs, making the data substantial. Fig. 1. Presents an overview of the dataset structure including the distribution of runs across the two failure modes, the repository file structure and an example representation of the measured variables and associated RUL.

The repository contains three main data components:

• “Extruder_Parameters.zip” contains the low-frequency process measurements for each run. These files are named following the format “RunX_extruder.csv” where X stands for the run number. Each “RunX_extruder.csv” file contains measurements sampled at 1 Hz, including temperatures along the barrel of the extruder, screw motor voltage and screw motor amperage. Each row corresponds to a 1 s timestep during the run.
• “Extruder_Current.zip” contains the high-frequency extruder current measurements for each run. These files are named following the format “RunX_current.csv” where X stands for the same run number used in the “RunX_extruder.csv” file. Each “RunX_current.csv” file contains the extruder electrical current draw sampled at 1 kHz. These files therefore provide millisecond-resolution current measurements for the corresponding run-to-failure trajectory.
• The “Labels.csv” file contains the metadata and labels for the 60 degradation runs. Each row corresponds to one run and includes the run identifier, the observed failure mode and the recorded lifetime of the run. The failure mode label is assigned at run level because each complete time-series corresponds to a single observed failure mode. The recorded lifetime can be used to derive RUL labels at different temporal resolutions. For a given run, the RUL at time t can be calculated as the recorded lifetime of the run minus the elapsed time, in seconds or milliseconds.

The sensor files are not provided with precomputed RUL labels at each time step. This format was chosen so that users can define prediction targets according to their modelling objective and preferred temporal resolution. For example, users may derive RUL targets as 1 s intervals using the 1 Hz process measurements or derive millisecond-scale RUL targets using the 1 kHz extruder current measurements. The same run-level labels can also be used for failure mode classification, where the target is the observed failure mode of the complete run. When both the failure mode and the derived RUL are used as targets, the dataset can be used for joint prognostic modelling.

## 4 Experimental Design, Materials and Methods

### 4.1 Experimental setup

**Fig. 2:**
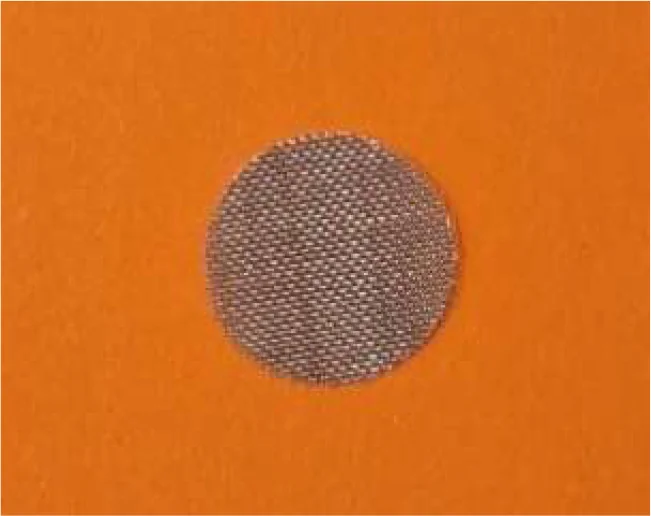
Example of a clean 80 opening per inch mesh filter.

The data was acquired on a Filabot EX6 industrial polymer extrusion system equipped with a melt filtration assembly in the die. The degrading component was the extruder filter (80 opening per inch mesh, 304 Stainless Steel) shown in Fig. 2, which was operated until end-of-life during repeated degradation experiments. Each experiment corresponds to a single run-to-failure trajectory with a new filter. A total of 60 run-to-failure experiments were recorded. The extrusion system is equipped with four independently controlled temperature zones, positioned along the extruder barrel: feed, back, mid and front. The front temperature zone extends to the filter-die assembly. The system was fitted with a standard EX6–625 screw with a compression ratio of 2:1 and an extended 1.75 mm filament die. The processed material was ExxonMobil® LLDPE 6101, produced by Saudi Aramco®.

The filter is installed in the die. During operation, the polymer melt passes through the filter before continuing through a breaker plate and exiting the die nozzle. As extrusion proceeds, contaminants accumulate on the filter. The filter is replaced between runs to prevent mechanical loading, deformation or thermal history from being carried over into the following run-to-failure.

The experiments were conducted in an industrial environment under normal operating conditions. The process was not isolated from industrial sources of electrical and mechanical noise. The recorded data therefore represents measurement conditions associated with routine operation of the extrusion system.

### 4.2 Run-to-failure experimental design

The run-to-failure experimental design was adapted from the methodology proposed by Schöppner & Meilwes [4] for the realistic and reproducible contamination of polymer extrusion melt filters. The chosen contaminant was ground calcite (CaCO_3_), sourced from Pacific Water Technology Pty LtD, Seventeen Mile Rocks, 4073, Australia. LLDPE granules were dried in a Piovan G31 for three hours before being contaminated with calcite at a concentration of 10 wt.% and the contaminated feedstock was homogenized before extrusion. The operating conditions under which the runs were conducted are available in Table 1. Experiments were conducted in laboratory conditions of temperature (approximately 21 °C) and pressure (atmospheric). The specific screw model employed for the experiment is a EX6–625, with a compression ratio of 2:1. Granule sizes are smaller than 3 mm. The hopper was gravity-fed, therefore, feed rate was not independently controlled or directly measured. To maintain consistent feeding conditions between runs, the same feedstock composition, screw-speed setpoint and initial hopper loading of 500 g were used. Prior to each degradation run, the extruder was brought to the specified temperature and screw-speed setpoints. Data associated with the degradation run were recorded after the steady-state operating conditions had been established, 10 min. During this time, uncontaminated feedstock material was used.

**Table 1:** Operating setpoints of the extruder.

Parameter	Screw Speed (rpm)	Feed T° (°C)	Back T° (°C)	Mid T° (°C)	Front T° (°C)
Value	12	35	160	180	190

The contaminated material was then processed through the extrusion system described in the previous section, with a clean filter installed before each run. All 60 experiments were conducted as continuous run-to-failure trials. No runs were interrupted and subsequently resumed, and no completed run-to-failure experiments were excluded from the released dataset. Consequently, each recorded sequence represents a continuous degradation trajectory from the beginning of the experiment to its observed terminal failure mode.

### 4.3 Process variables acquisition

**Fig. 3:**
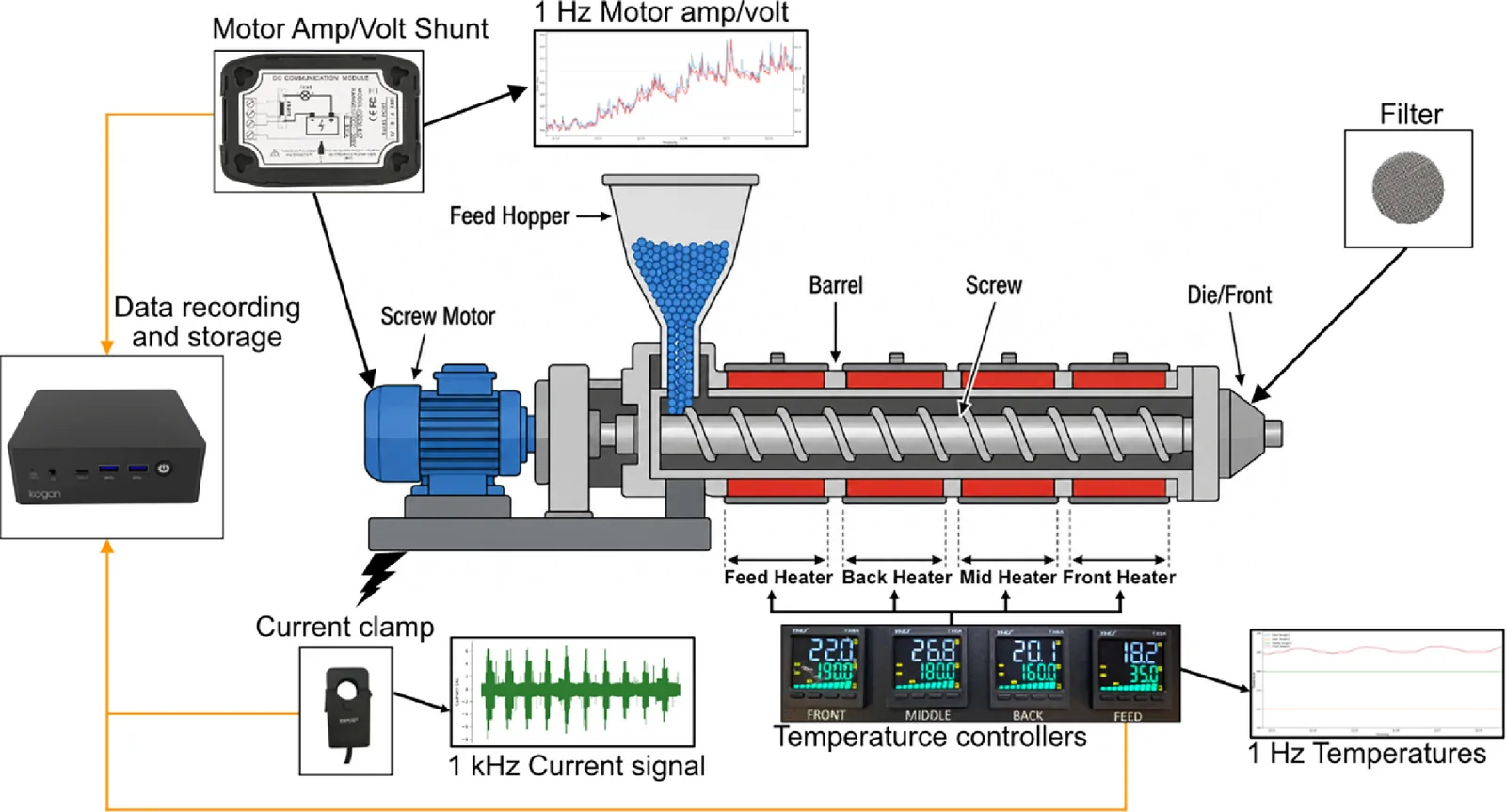
Dataset collection setup.

A high-level view of the data acquisition setup is provided in Fig. 3 and details are provided in Table 2. Low-frequency process variables were acquired from the extrusion system at a sampling frequency of 1 Hz. Temperature measurements were acquired from each zone using YHKJ TX6-AK220RS485 PID controllers equipped with RS-485 communication ports. Each controller is connected to a Type-K thermocouple screwed into the barrel at each corresponding temperature zone. The type-K thermocouples were calibrated before being installed by comparing the voltage output at reference temperature points with a reference table.

**Table 2:** Measurement and Data Acquisition System Specifications.

Variable	Sensor	Range	Accuracy	Resolution	Sampling	Interface
Motor Volts	PZEM-017	0.05–300V	1%	0.01V	1 Hz	RS485
Motor Amps	PZEM-017 with shunt	0.02–50A	1%	0.01A	1 Hz	RS485
Temperatures (Feed-to-Front)	Type-K Thermocouple (brand: Phipps)	0⁰C −700⁰C	0.5%	0.1⁰C	1 Hz	YHKJ TX6 PID + RS485
Extruder Current	YHDC HTS016L	±15A	1%	78 mA	1 kHz	10bit ADC

Screw motor voltage and amperage were collected at 1 Hz using a PZEM-017 electrical submeter with an integrated RS-485 communication port. The PZEM-017 was calibrated using reference points obtained by analog readings of known voltage and current.

Extruder current draw was acquired at a sampling frequency of 1 kHz using a YHDC HTS016L current clamp connected to the live conductor supplying the extruder. The current clamp originally had an offset, which was removed by calibrating against a known zero current value.

All signals are recorded and converted into measurements using Python scripts running on a Kogan Atlas G900 Mini, stored in an InfluxDB database. 1 Hz process variables and 1 kHz extruder current data are recorded separately and exported as .csv files for each run. A data quality audit is available in Table 3, in which we describe missing values, duplicate rows, timestamp irregularities, clipped segments and interrupted runs. Timestamp regularity was evaluated from consecutive timestamp differences relative to the nominal sampling interval of 1 ms or 1 s.

**Table 3:** Data Quality Audit.

Data	Nominal Frequency	Nb. of Samples	Missing Samples	Duplicate Samples	Irregular Timestamps	Clipped Samples	Interrupted Runs
High-Frequency Current	Nominal 1 kHz	27,801,000	0	4572	16 797	0	0
Process Variables	1 Hz	27 801	0	0	326	0	0

### 4.4 Failure-mode and lifetime labelling

**Fig. 4:**
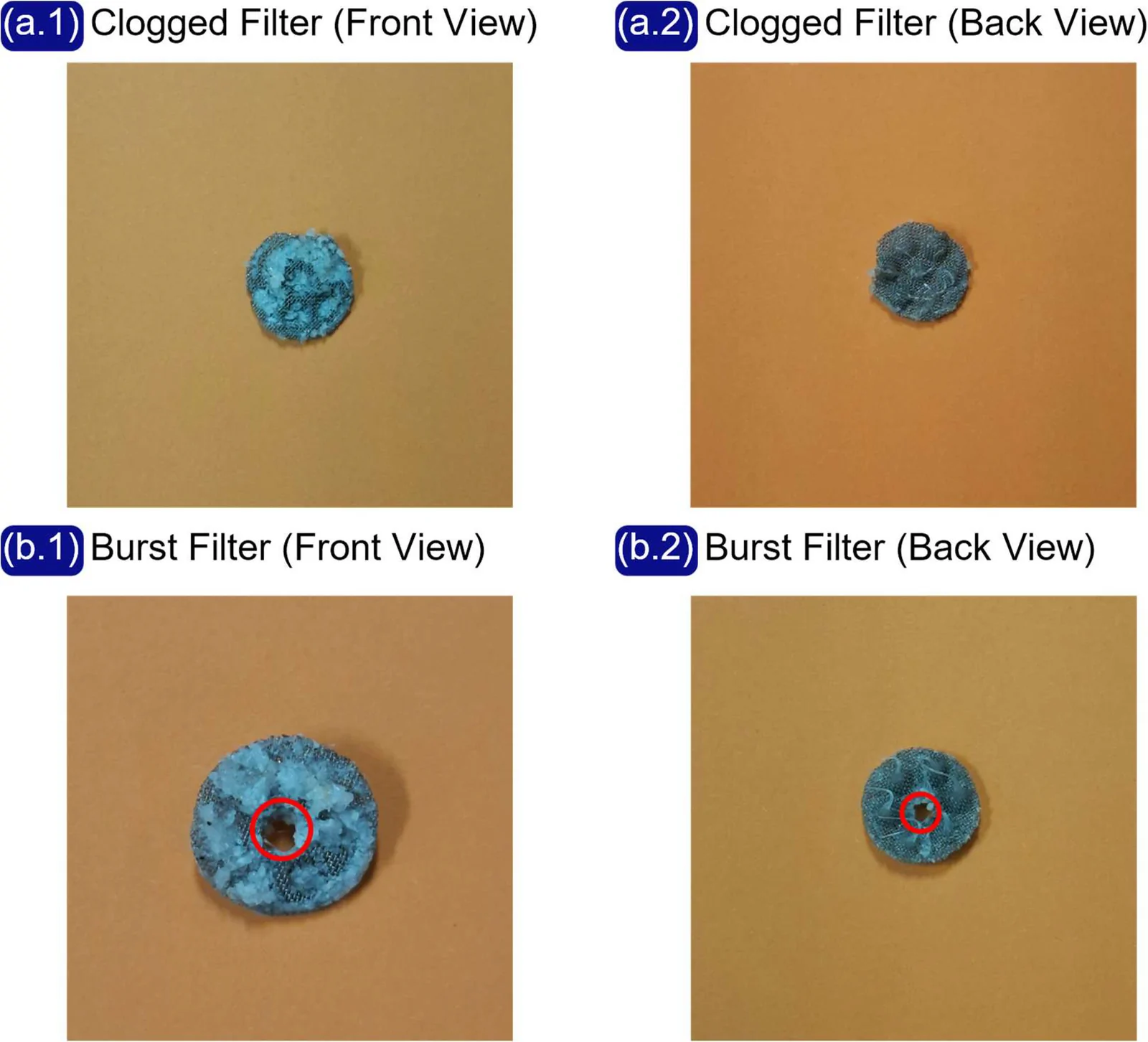
Examples of clogged and burst filters. (a) Clogged filter. The white contaminants accumulate on the front section, facing the flow. (b) Burst filter. Circled in red is the burst area.

The failure label was assigned after the end-of-life event of each run. A run was labelled “clog” when polymer material stopped exiting the die, indicating that material flow through the filter and die assembly had ceased. Additionally, filter inspection was carried out to confirm no damage. During a clog, the contaminant accumulates onto the filter, which causes a progressive reduction in the available filter area, thus an increased resistance to melt flow, and consequently an increased mechanical loading due to the filter obstruction. A run was labelled as “burst” when contaminants were observed in the extruded material after passing through the die and confirmed by post-run inspection of the filter. The burst is caused by an increase of the mechanical load onto the filter, which progressively deforms or ruptures the filter mesh. A filter opening gets progressively enlarged, and as it becomes the path of least resistance, contaminated melt rushes through the opening. Examples of filters corresponding to “clog” and “burst” are shown in Fig. 4. The recorded lifetime corresponds to the elapsed time from the start of the run to the recorded failure time. The data collection continued past one of the failure modes had become visually noticeable (complete absence of flow or change in flow and presence of contaminants) and was stopped 10 s after the event. The corresponding electrical current signature of the screw motor was inspected to identify the timestamp associated with the failure of the filter. For the clog failure, the motor current increases and then plateaus as the filter reaches a fully clogged state. The transition point between the increasing-current region and the plateau was used as the failure timestamp. For the burst failure, the motor current increases to a maximum, and as the burst occurs, drops rapidly. Identifying the turning point from increase to decrease of the motor current provides a timestamp for the burst failure.

While specific RUL normalization, modelling strategy, evaluation metrics and treatment of competing failure modes are intentionally not prescribed as they depend on the research question of the users, some guidelines to prevent misuse are provided in Table 4.

**Table 4:** Dataset reusability considerations and protocols.

Prognosis Aspect	Dataset reuse consideration
Independent experimental units	Samples or windows extracted from the same trajectory are temporally dependent and should not be treated as independent observations. A train/test split per run is recommended.
Data partitioning	Specific train/validation/test proportions are left to the users, a cross-validation is recommended considering 60 runs are available.
RUL construction	Each trajectory contains timestamps. It is recommended to construct absolute RUL targets: $\text{RUL}\left(t\right)=T_{\text{final}}-t$. Alternative normalization or target formulations is selected according to the intended prognostic method.
Failure modes	Each run terminates in either clogging or filter burst. These labels are provided as metadata. They may be treated as separate classes (for classification using time-series) or incorporated into prognostic formulations, depending on the research question.
Sequence lengths	Trajectory lengths vary because experiments were continued until the observed failure mode occurred. Padding, truncation, windowing, resampling or other sequence-handling methods are left to the user.
Performance evaluation	The dataset does not prescribe specific metrics. Users should select evaluation measures appropriate to their modelling objective. It is important that the class imbalance is reflected in the metrics choice.

### 4.5 Data acquisition, timing and synchronization

The process variables and high-frequency current measurements were obtained through separate acquisition paths. The process variables were polled by an Arduino card at a nominal 1 Hz frequency using an internal elapsed-time routine based on the microcontroller clock. The high-frequency current-clamp signal was acquired separately using an analogue current-clamp interface and dedicated Arduino microcontroller due to the high-frequency rate that would have otherwise saturated the process variable Arduino. Measurements were transferred through a serial connection and recorded to an InfluxDB timeseries database. Individual high-frequency measurements were assigned host-system timestamps before database submission. Timestamps were generated using the system clock. No external synchronization trigger was shared between the low- and high-frequency acquisition paths. The high-frequency channel is consequently described as nominally sampled at 1 kHz. No dedicated correction for relative clock drift between the acquisition systems was implemented and serial-transfer and operating-system scheduling latencies were not independently characterized. For multi-rate analysis, users should use the supplied temporal information and corresponding experimental-run identifiers to establish temporal correspondence. The data are suitable for run-scale alignment and multi-rate feature extraction, but millisecond-level phase synchronization between the 1 Hz and nominal 1 kHz channels should not be assumed. During the data quality audit, it was identified that the maximum timestamp mismatch across all runs was 36 ms.

## Limitations

The dataset was generated under controlled experimental conditions which present several limitations. First, experiments were conducted using a single polymer material, screw geometry and contaminant type. This controlled configuration supports repeatability but limits the ability to evaluate generalization across different materials, screw designs, contaminant types and associated operating conditions. Second, failure mode labels were assigned after each run based on inspection criteria. Clogging was labelled when material flow stopped at the die outlet. Burst events were labelled when visible contamination was observed in the extrudate and labels were subsequently confirmed by a post-run inspection of the filter. A clogged filter is structurally intact but there is a high amount of contaminant, while a burst has an identifiable rupture. As these labels relied partly on visual assessment, some subjectivity or human error may be present. Third, although the contaminant concentration was defined before each run and homogenized with the material, the spatial homogeneity of contaminant dispersion within the melted material in the extruder barrel was not measured. Consequently, local variations in contaminant distribution may have affected failure progression and the recorded lifetime and the nominal wt.10% value describes the prepared mixture and should not be interpreted as a verified instantaneous contaminant concentration at the filter. The contaminated material was then processed through the extrusion system described in the previous section, with a clean filter installed before each run. The filters employed conform to the ASTME2016 norm, which imposes a maximum 2% tolerance on warp weft directions. While this variability is common, it makes the experiment less controllable. Finally, the dataset comprises 60 run-to-failure experiments. Although this represents a substantial experimental effort for a controlled degradation study, the number of independent failure trajectories remains limited for training data-intensive models and is missing additional measurements such as melt and die-filter assembly pressures, which could not be conducted due to the compactness of the die-filter assembly. However, screw-motor measurements are related to motor torque and physically correlated with mechanical loading, providing a proxy indicator of the missing pressure measurements.

From a methodological perspective, the effective sample size of the dataset is the 60 independent run-to-failure trajectories. For machine-learning applications, train/validation/test partitioning should therefore be performed at the trajectory level rather than by randomly splitting individual samples or windows, as the latter may place observations from the same run in multiple subsets and introduce information leakage. Users should also account for the imbalance between the two terminal outcomes and their different lifetime distributions when designing and evaluating predictive models.

## Ethics Statement

Authors confirm that they have read and follow the ethical requirements for publication in Data in Brief and confirm that the current work does not involve human subjects, animal experiments or any data collected from social media platforms.

## Credit Author Statement

**Corentin Ferreira:** Data Curation, Methodology, Writing – Original Draft; **Ben Ko:** Data Curation, Methodology; **Wen Li:** Supervision; **Kevin Otto:** Supervision, Writing – Review & Editing.
